# Salivary ammonia levels and Tannerella forsythia are associated with rheumatoid arthritis: A cross sectional study

**DOI:** 10.1002/cre2.68

**Published:** 2017-06-07

**Authors:** José‐Iván Martínez‐Rivera, Daniel X. Xibillé‐Friedmann, Judith González‐Christen, Myriam A. de la Garza‐Ramos, Sandra M. Carrillo‐Vázquez, José‐Luis Montiel‐Hernández

**Affiliations:** ^1^ CISEI Instituto Nacional de Salud Pública Cuernavaca Morelos 62100 Mexico; ^2^ SEIC-Servicios de Salud de Morelos Mexico; ^3^ Facultad de Farmacia Universidad Autónoma del Estado de Morelos Cuernavaca Morelos 62209 Mexico; ^4^ CIDICS/Facultad de Odontología Universidad Autónoma de Nuevo León Monterrey Mexico; ^5^ Hospital Regional 1o. Octubre ISSSTE Mexico City Mexico

**Keywords:** periodontitis, rheumatoid arthritis, T. forsythia, salivary ammonia levels

## Abstract

The aim of this paper is to evaluate the relationship of salivary ammonium levels and the presence of bacteria with rheumatoid arthritis (RA) clinical disease activity in a cross‐sectional study of Mexican patients. From a periodontal and disease activity standpoint, 132 consecutive RA patients fulfilling clinical criteria were evaluated. Ammonia levels (including peptidyl arginine deiminase activity) were evaluated by colorimetric assay and the presence of *Porphyromonas gingivalis*, *Tannerella forsythia*, and *Prevotella intermedia* was evaluated by polymerase chain reaction (PCR) technique. After a multivariate analysis, adjusting for clinical and serological parameters, a significant association was only observed between severe periodontitis and probing depth with high RA disease activity. Additionally, in contrast to *P. gingivalis*, the presence of *T. forsythia* was significantly associated with high disease RA activity even after multivariable adjustment analysis. There was also a significant increase in ammonium levels in the high RA activity group and a significant correlation between salivary ammonia and RA disease activity but not with autoantibody titers. Similarly, we observed a significant increase in the ammonium levels derived from the cultures of *P. gingivalis* and *T. forsythia*, with respect to *P. intermedia* and *S. gordonii* cultures, or even healthy donors. These results suggest that RA activity is associated with severe periodontitis, high salivary ammonium levels and the presence of *T. forsythia*.

## 1 INTRODUCTION

Rheumatoid arthritis (RA) is a chronic autoimmune disease characterized by inflammation, structural damage, and loss of function of the synovial joints. Its etiology is unknown but it is associated with several factors (Smolen, Aletaha, & McInnes, 2016), and recent studies suggest that one of those involved in RA development and disease activity could be severe periodontitis (PD); (Chen et al., 2013, Konig, Paracha, Moni, Bingham, & Andrade, 2015, Leech & Bartold, 2015, Payne, Golub, Thiele, & Mikuls, 2015, Venkataraman & Almas, 2015). It is well established that oral microbiome disruption in gingival pockets leads to PD, an oral chronic disease characterized by inflammation and destruction of dental support tissues. It is also known that certain bacterial strains, such as *Porphyromonas gingivalis*, *Tannerella forsythia*, and *Prevotella intermedia,* are associated with PD (Darveau, 2010, Mihai et al., 2015, Walker, 2004). In particular, *P. gingivalis* has been associated with the development of RA, mostly by the characteristic expression of a secreted‐form of peptidyl arginine deiminase (PAD; *P. gingivalis*, PPAD), capable of citrullinating host proteins and potentially favoring the development of autoantibodies. (Konig et al., 2015, Scher & Abramson, 2011, Wegner et al., 2010). Although *P. gingivalis* seems critical in explaining the potential effects of severe PD in the development of RA, its association with the presence of the bacteria or anti‐*P. gingivalis*/anti‐PPAD antibodies is not always evident (Konig et al., 2015, Scher et al., 2012, Ziebolz et al., 2011). Moreover, global periodontal microbiome studies done in patients with early‐onset RA do not show a potential relationship with *P. gingivalis*, but with other germs (*Anaeroglobus geminatus*) or species from the *Prevotella* and *Leptotrichia* genus (Scher et al., 2012).

On the other hand, the levels of ammonium in saliva, among other factors, depend on the passive diffusion of ammonium from the blood to the salivary glands and from the metabolism of arginine and urea by the oral bacteria. Because the enzymatic determination of ammonium can be coupled with the citrulination activity by PAD, we wanted to know whether it is possible to observe differences between patient groups and bacterial cultures.

In addition, association studies between RA and PD have led to heterogeneous results, probably because the variables have not been similarly controlled or clinical outcomes have been different in each case. Therefore, the objective of this study is to confirm the association between RA inflammatory disease activity in a homogeneous population positive to anti‐cyclic citrullinated peptide (αCCP^+^) antibodies, oral PAD activity, and the prevalence of previously incompletely evaluated bacterial strains, such as *T. forsythia*.

## 2 MATERIALS AND METHODS

### 2.1 Population and samples

Consecutive patients were recruited at the rheumatology outpatient clinic of the Hospital General de Cuernavaca in Morelos, Mexico, between July 2014 and June 2015. The cross‐sectional study protocol was reviewed and approved by the institutional (INSP) and hospital ethics' committee. Signed informed consent was obtained from all patients during recruitment. All patients received combination treatment with disease‐modifying antirheumatic drugs, mainly methotrexate and cloroquine and, in some cases, 10 mg or less per day of prednisone. No patients were treated with biologics. All patients included were seen consecutively and classified as having RA per the American College of Rheumatology/European League Against Rheumatism (ACR/EULAR) 2010 criteria (Aletaha et al., 2010). Disease activity was measured using a three‐variable index DAS28 (swollen and tender peripheral joints and the erythrocyte sedimentation rate [ESR], DAS28‐ESR) and was performed by the same rheumatologist (DXXF). Subsequently, the clinical activity index used in this study DAS28‐ESR will be reported only as DAS28. For the comparative analysis, patients were stratified per DAS28 activity into the following groups: low disease activity (DAS28 remission to 3.2) and high disease activity (DAS28 > 3.2). All patients were αCCP positive and had a complete medical history on file. Patients were excluded if they had an uncontrolled systemic condition such as diabetes or hypertension or some other autoimmune disease, as well as if they had been diagnosed with Sjögren's disease or sicca‐syndrome, if they were active smokers and/or if they had received periodontal treatment (antibiotics) in the year prior to the sampling or had chronic liver disease. On the day of the clinical evaluation, patients provided venous blood and saliva samples; these were then processed on the same day and frozen at −80 °C.

### 2.2 Periodontal evaluation

As part of the clinical evaluation, RA patients were subject to periodontal examination and testing. To determine the periodontal status, the following parameters were recorded: probing depth, clinical degree of tooth attachment, and bleeding upon probing. The periodontal status in all patients was determined according to the criteria proposed by the American Dental Association using a periodontal probe suggested by the World Health Organization (Wiebe & Putnins, 2000) and classified as follows: mild PD (colored area of the probe remains completely visible at the deepest probing depth and supra or subgingival calculus and/or defective margins are detected in at least one sextant) and severe PD (colored area of the probe completely disappears indicating a probing depth between 3.5 to 5.5 mm in at least one sextant). Gingivorrhagia was determined as the presence of spontaneous gum bleeding at probing. Dental mobility was determined in the number of millimeters that teeth moved in alveolar bone and dental loss was determined by the presence or absence of dental organs. Cementum‐enamel junction was evaluated as previously described (Eley & Cox, 1998). All periodontal evaluations were done by the same person (JIMR).

### 2.3 Antibody determination

αCCP titers were determined using a commercial anti‐cyclic citrullinated peptide enzyme‐linked immunosorbent assay (*Euroimmun*) following the procedures recommended by manufacturer. Patients were considered as αCCP positive if the titers were 5 RU/ml or greater. Rheumatoid factor (RF) was qualitatively evaluated by nephelometry. Relative abundance of circulating immunoglobulin G (IgG) anti‐*P. gingivalis* antibodies were obtained by indirect enzyme‐linked immunosorbent assay, using the whole bacterium (strain W83) as a reference and anti‐Human IgG‐HRP for detection (Lee et al., 2015).

### 2.4 Enzymatic determination of salivary ammonium levels

As a quantitative approximation of the contribution of bacterial PAD to the oral microenvironment in patients with RA, we used a highly reproducible enzymatic method, which is based on the determination of ammonium levels either by the action of bacterial PAD or ammonium already present in the saliva sample.

Total salivary ammonium levels were determined as follows: a 1.0 μg of saliva protein (determined by Bradford's technique) was diluted in 50 μl of deionized water (Direct‐Q5UV‐R, MilliPore). Samples were incubated in 150 μl tris‐base solution (200mM) pH 9, 8.5mM of α‐ketoglutaric acid (Sigma), 0.22mM of reduced β‐Nicotinamide adenine dinucleotide (Sigma), and 3U of L‐Glutamic Dehidrogenase (Sigma); a final volume of 200 μl was placed in 96 wells (Corning UV plate). Additionally, 10 mM of benzoyl (Bz)‐L‐Arg‐ethyl‐ester (BAEE, arginine rich substrate) was also included to the contribution of PAD activity. Ammonium levels were considered as referenced to the NADH oxidation, as described previously (Liao, Hsieh, Liu, & Hung, 2005). Velocity was visualized at 340 nm in a spectrophotometer (Epoch, Biotek) for 20 min with an interval of 2 min at 24 °C and determined using the following formula: Vel (mM/min/μg) = (OD_i_‐OD_f_/20)/ε NADH(0.00622M)

Additionally, assays employing salivary samples from healthy donors (*n* = 10) free from rheumatic and periodontal diseases were employed as a reference of baseline ammonium salivary levels.

### 2.5 Identification of oral bacteria

With the objective of evaluate if *Tanerella forsythia* could be associated to RA or oral PAD activity, we compared its presence with respect other bacteria species recognized as associated (*Porphyromonas gingivalis)* and non‐associated *(Prevotella intermedia)* to RA development (Mikuls et al., 2012, Wegner et al., 2010). All evaluations were performed using end‐point PCR. Samples were obtained from decanted saliva (~5 ml) in a 15‐ml sterile tube directly suspended in a protease‐inhibitor (EASYpack, ROCHE). DNA was extracted by TRIZOL (Invitrogen) as previously described (De La Garza‐Ramos, Galan‐Wong, Caffesse, Gonzalez‐Salazar, & Pereyra‐Alferez, 2008). Samples were stored at ‐75 °C. Specific bacterial 16S ribosome primers were *P. gingivalis* forward, AAGGATTGTAAACTTCTTTTATAC; reverse, ACTGTTAGCAACTACCGATGT (*Genebank POYRR16SC*); *T. forsythia* forward, GCGTATGTAACCTGCACCTGCCCGCA; reverse, GAAGGCAGCTTACTAAGG (*Genebank* BFORR16S); *P. intermedia* forward, GCATTTACCCTTCGAATAAGGACC; reverse, GAGTCAACATCTCTGTATCCTGCG (*Genebank* PVORR16SD); PCR conditions were denaturing 95 °C 30 cycles/5 min; annealing 55 °C/30 s; extending 72 °C/10 min, in a thermocycler (TECHNE^3^prime). PCR analysis was performed using 2% agarose gel in TAE (tris‐acetate‐EDTA)/100mV/10 min; densitometry was performed using a ChemiDoc XRP (Bio‐Rad) transilluminator with Quantity One v.4.6.6 software. Presence of bacterial strains was considered positive if the density bands showed a 10‐fold intensity (INT*mm^2^) or greater with respect to gel background.

### 2.6 Statistical analysis

Descriptive statistics were employed for continuous variables. Multivariate analysis was done adjusting for gender, age, time since onset of disease, RF, and ESR. Comparisons between groups were performed using Mann–Whitney U testing, because experimental values did not show a normal distribution. Kruskal–Wallis testing was performed to compare patient and bacterial PAD activity. Spearman correlation testing was employed to correlate PAD activity and DAS28. Statistical significance was set at .05% (STATA v.13).

## 3 RESULTS

Initially, 208 RA patients were invited to participate in the study but only 132 RA patients were positive to αCCP (αCCP^+^) and were included in this analysis; of these, 129 were female. According to their RA activity, patients were divided into two subgroups: 36 with low disease activity (DAS28 ≤ 3.2) and 96 with high disease activity (DAS28 > 3.2, Table 1). We did not find statistical differences between subgroups regarding gender, age, or time since onset of disease. Statistically, significant differences between groups were found in αCCP titers (*p* = .001), ESR (*p* < .001), and RF positivity (*p* < .01). No statistical differences in anti‐*P. gingivalis* IgG‐antibody titers were observed between groups.

**Table 1:** Clinical and serological characteristic of RA patient groups

	Low activity (DAS28 ≤ 3.2) *n* = 36	High activity (DAS28 > 3.2) *n* = 96	*p*
Gender (Male/Female), n	1/35	2/94	
Age, years, (*SD*)	46 (9.4)	47.5 (11.1)	
Years since onset of RA, mean (*SD*)	3.0 (4.5)	8.4 (7.8)	
DAS28, mean (*SD*)	2.2 (1.4)	4.6 (0.9)	.001**
αCCP, UR/mL	6.4 (17.4)	139.9 (55.2)	.001*
ESR, mm/h, mean (*SD*)	10.4 (2.8)	30.8 (1.3)	.001*
RF, n (% positive)	9 (25.0)	93 (96.8)	.01**
IgG anti‐*P. gingivalis*, AU (*SD*)	4.5 (3.2)	3.8 (0.72)	.68*

*Note*. αCCP = anti‐cyclic citrullinated peptide; ESR = erythrocyte sedimentation rate; RA = rheumatoid arthritis; *SD* = standard deviation.

* Mann–Whitney test, *p* < .05.

** Pearson chi^2^, *p* < .05%.

A baseline association analysis (Table 2) between PD and RA determined that severe PD (OR, 2.8 [1–16.8]), gingivorrhagia (OR, 2.7 [2.0–12.2]), dental mobility (OR, 2.1 [1.8–5.6]), and probing depth (OR, 5.3 [1.6–7.1]) had a significantly higher OR in the highly active RA patients. In an ulterior multivariable analysis, after adjusting for different parameters (age, time since onset of RA, ESR, and RF), only the group of high RA activity showed an association to severe PD (OR, 3.3 (2.8–13.6), *p* < .004) and probing depth (OR, 4.8 (1.4–8.7), *p* < .009). For their part, gingivorrhagia and dental mobility lost its high OR after adjusting for ESR. Other periodontal parameters were not associated with high RA activity either in the baseline analysis or after the adjustment of parameters. Based on the sample size of the groups classified in high and low DAS28, a statistical power of 98% was determined.

**Table 2:** Association analysis of periodontal characteristics between RA patient subgroup

DAS28 vs		*p*	Adjusted^a^ OR (95% IC)	*p*
Severe periodontitis				
Low activity	1.6 (0.8–3.4)	.16	1.0 (1.0–1.1)	.09
High activity	**2.9 (2.0**–**9.3)**	**.001** *	**3.3 (2.8**–**13.6** **)**	**.004** *
Gingivorrhagia				
Low activity	1.7 (2.0–15.1)	.09	1.1 (0.6–17.9)	.09
High activity	**2.7 (2.0**–**12.2)**	**.003** *	1.6 (0.9–2.9)	.05
Dental mobility				
Low activity	0.72 (0.9–5.7)	.7	0.32 (0.015–9.4)	.17
High activity	**2.1 (1.8**–**5.6)**	**.003** *	1.5 (0.81–1.3)	.07
Dental loss				
Low activity	0.83 (0.25–2.6)	.7	0.33 (0.3–2.4)	.23
High activity	1.9 (0.55–6.5)	.3	2.0 (0.4–4.9)	.37
Probing depth				
Low activity	0.53 (0.11–2.4)	.45	0.46 (0.99–2.1)	.32
High activity	**5.3 (1.6**–**7.1)**	**.005** *	**4.8 (1.4**–**8.7)**	**.009** *
CEJ^a^				
Low activity	0.85 (0.21–3.4)	.83	0.97(0.21–4.3)	.63
High activity	1.7 (1.01–5.6)	.06	2.0 (0.15–5.9)	.08
*P. gingivalis*				
Low activity	1.6 (0.5–5.2)	.42	1.7 (0.49–6.4)	.37
High activity	**5.3 (1.8**–**6.1)**	**.002** *	5.3 (4.8–5.9)	.05
*T. forsythia*				
Low activity	2.4 (0.7–8.0)	.14	1.4 (0.9–1.9)	.78
High activity	**5.3 (1.4**–**6.3)**	**.001** *	**4.4 (2.5**–**5.3)**	**.002** *
*P. intermedia*				
Low activity	0.68 (0.16–2.7)	.59	0.99 (0.96–1.0)	.95
High activity	0.83 (0.26–2.6)	.75	0.34 (0.07–1.6)	.18

*Note*. ESR = erythrocyte sedimentation rate; RA = rheumatoid arthritis; RF = rheumatoid factor.

Adjusted by age, time since onset of RA, ESR, and RF.

a Cementum‐enamel junction. Statistical analysis, chi^2^ Pearson.

* *p* < .05.

The use of bold in Table is to increasing visualization but in the actual Table's format is not necessary to conserve the bold font.

The lower half of Table 2 shows the association analysis between the frequency of bacterial strains (*P. gingivalis*, *T. forsythia*, and *S. intermedia)* and disease activity subgroups. In the baseline analysis, a significant association was observed between high RA activity and the presence of *P. gingivalis* (OR, 5.3 (1.8–6.1), *p* < .002) or the presence of *T. forsythia* (OR, 5.3 (1.4–6.3), *p* < .001). After statistical adjustment, a significant association with the high RA activity group remained only for *T. forsythia* (OR, 4.4 (2.5–5.3), *p* < .002). As before, the baseline association between the high RA activity group and the prevalence of *P. gingivalis* disappeared when adjusting for ESR. As expected (Wegner et al., 2010), presence of *P. intermedia* did not show an association with any RA disease activity group or baseline data.

**Figure 1:**
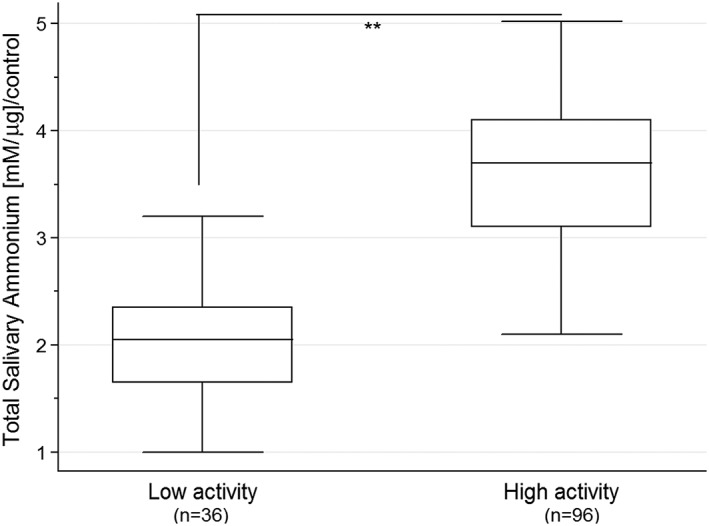
Increased levels of total ammonium were observed in salivary samples from high RA activity patients, in comparison to low RA activity. Enzymatic assay for total ammonium levels determination is described in Methods. Graphical analysis was done normalizing for experimental control (without oral samples). Statistical comparison was done employing Mann–Whitney test, considering *p* < .01 (**)

**Figure 2:**
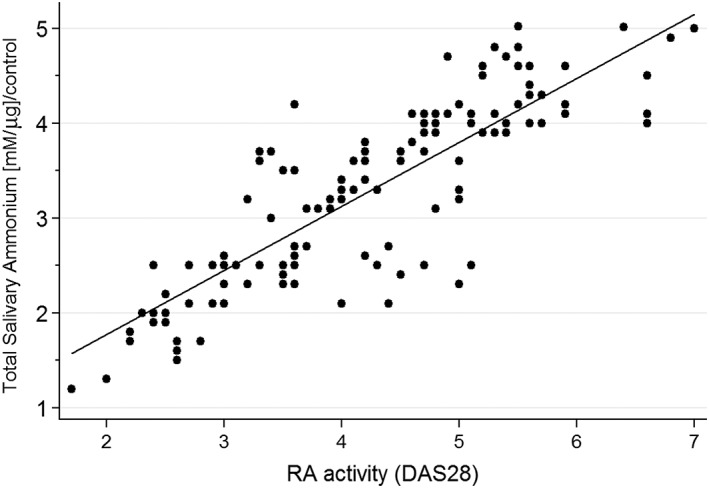
Total salivary ammonium levels positively correlated with RA clinical activity. Values from all RA patient subgroups were correlated (Spearman) with clinical activity (DAS28), showing ρ = 0.67 and *p* < .001

Comparing total ammonium levels in saliva samples of RA patients, we found (Figure 1) significant differences between the low (1.995 ± 0.48) and high (3.611 ± 0.76) RA disease activity groups (*p* < .01%). We did not see statistical differences in saliva ammonium levels between healthy donors and low RA activity patients (Figure S1), whereas high activity patients showed a 180% increase in ammonium levels. Because ammonium levels seemed to vary between RA disease activity subgroups, a correlation analysis for all RA patients was carried out. Figure 2 shows a significant correlation (ρ = 0.67, *p* < .001) between total ammonium levels and DAS28, suggesting a biological link between salivary environment and the RA disease activity. On the contrary, titers of aCCP antibodies showed no statistical correlation with total ammonium levels in saliva samples (data not shown).

Because our assay did not distinguish the contribution of the enzymatic activity of PAD and ammonium already present in the saliva sample, these results were compared with those obtained when using individual cultures of the *P. gingivalis*, *T forsythia*, *P. intermedia*, *and S. gordonii* strains (Table 3). The results showed that total ammonium levels were practically undetectable in *P. intermedia* and *S. gordonii*, but an increase of approximately 1,300% was observed when compared with to *P. gingivalis* or *T. forsythia* cultures, suggesting that some bacterial products characteristic of *P. gingivalis* or *T. forsythia* cultures are responsible for the generation of high levels of ammonia in our enzymatic assay.

**Table 3:** Comparative total ammonium levels^a^ in culture media of bacteria strains

*P. gingivalis*	*T. forsythia*	*P. intermedia*	*S. gordonni*
m (*SD*)	m (*SD*)	m (*SD*)	m (*SD*)
4.0 (0.06)	3.9 (0.02)	0.1 (0.17)	0.04 (0.23)

[mM/μg]/control.

Table 4 shows that this effect increases linearly with the increase in protein concentration and has an additive character between the cultures of both bacterial strains. This suggests that the presence of at least these two bacteria contribute equally to alter either the level of citrullination or the levels of ammonium in saliva, and these events relate to the clinical activity of the patients with RA. On the other hand, and in line with a higher prevalence of *P. gingivalis* or *T. forsythia* in the subgroup with severe PD, we confirmed (Figure S2) that PAD activity was significantly lower in the mild PD subgroup in comparison to the severe subgroup (*p* < .001).

**Table 4:** Total ammonium levels^a^ by soap culture of *T. forsythia* or *T.f.* + *P.g*

0.1 [μg/μl] m (*SD*)	0.5 [μg/μl] m (*SD*)	1.0 [μg/μl] m (*SD*)	*T. forsythia* [1.0 μg/μl] + *P. gingivalis* [1.0 μg/μl] m (*SD*)
0.9 (0.15)	3.3 (0.13)	5.4 (0.37)	10.8 (0.32)

*Note*. *P.*g = Porphyromonas gingivalis; *T.f* = *Tannerella forsythia*; *SD* = standard deviation.

a [mM/μg]/control.

## 4 DISCUSSION

Several previous studies have suggested an association between severe PD with RA; however, due to differences in the classification of PD as well as the control of parameters, the results have been heterogeneous (Lee et al., 2015, Mikuls et al., 2014, Scher et al., 2012, Seror et al., 2015, Ziebolz et al., 2011). In this respect, the lack of association between the prevalence of *P. gingivalis* or anti‐*P. gingivalis* antibodies (e.g., in this study) with relation to RA raises important questions about their participation in the autoimmune process (Scher et al., 2012, Ziebolz et al., 2011). Furthermore, recent studies have suggested that periodontal treatment alone can generate positive effects in the clinical manifestations of RA patients (Biyikoglu et al., 2013, Okada et al., 2013, Roman‐Torres et al., 2015); however, these studies have been conducted with small numbers of patients and lack a rigorous evaluation (Kaur, Bright, Proudman, & Bartold, 2014).

On the other hand, there is ample evidence that seems to coincide with the fact that the oral microenvironment may cause periodontal bacteria‐mediated chemical modification of connective tissue proteins, present within the joints of RA patients (Venkataraman & Almas, 2015). In this regard, it has been confirmed that the peptidyl arginine deiminase expressed by *P. gingivalis* (PPAD), despite having no homology to the PADs present in mammals, leads to citrullination of human proteins (Konig et al., 2015, Montgomery et al., 2016, Rodriguez, Stitt, & Ash, 2009, Wegner et al., 2010). Furthermore, active participation of PPAD was also confirmed in a collagen induced arthritis model, suggesting a biological connection between *P. gingivalis* infection and RA (Maresz et al., 2013). On the other hand, a comparative study with 10 other oral bacteria suggested that *P. gingivalis* was the only one capable of carrying out this enzymatic reaction (Wegner et al., 2010); however, *T. forsythia* was not included in this study. Additionally since then, knowledge on the periodontal microbiology has been enriched and compiled into the human oral microbiome (Cross, Faustoferri, & Quivey, 2016); the genome of *T. forsythia* (Friedrich et al., 2015) in this database has recently been corrected, allowing for the verification of previous studies.

The present study confirms the existence of a significant association between severe PD and probing depth with RA, as previous studies have done (Chou, Lai, Chen, Lin, & Chen, 2015, de Smit et al., 2012, Fuggle, Smith, Kaul, & Sofat, 2016, Mikuls et al., 2014); however, no association was observed with gum bleeding or tooth loss (although an association was seen in the bivariate analysis, this was not observed in the multivariate analysis, probably due to the sample size, and should be explored further), as others have suggested (Demmer, Molitor, Jacobs, & Michalowicz, 2011, Kobayashi et al., 2010, Torkzaban, Hjiabadi, Basiri, & Poorolajal, 2012). Importantly, this association remained even after adjusting for clinical and serological parameters, suggesting an independent relationship between PD and RA. A previous study within the same cohort, which evaluated the possible clinical association of treatment response in RA patients with levels of leptin or adiponectin, as well as overweight and obesity showed an association of circulating leptin and clinical activity (Xibille‐Friedmann et al., 2015), but that was not the case in the present study because differences in the body mass index between patients did not change the association values of PD or oral PAD activity (data not shown). Similarly, pharmacological treatment appears to not have influenced the results because more than 80% of patients remained within the general treatment regimen (MTX + chloroquine + prednisone incidentally). Also, no reports were found suggesting side effects of antirheumatic treatment on the oral microenvironment.

For its part, it is important to note that the association of *T. forsythia* with the high RA activity group remains even after adjusting for clinical or serologic parameters (OR, 4.4 (2.5–5.3), p < .002), suggesting a potential role of the bacteria in the RA activity, similar to what was proposed for *P. gingivalis* (Konig et al., 2015). On the other hand, and similarly to other studies (Konig et al., 2011, Scher & Abramson, 2011, Ziebolz et al., 2011), we did not observe a significant association between *P. gingivalis* and high RA activity when performing the multivariate analysis; in fact, after adjusting for ESR, the association observed in the bivariate analysis was lost (Table 3). Finally, the presence of *P. intermedia* was not associated at all with disease activity. It is well known that these three bacteria commonly occur together in the same periodontal inflammatory process, and there is ample biological evidence that confirms their interrelationship (Endo et al., 2015, Ng et al., 2016, Zhu & Lee, 2016). In this sense, it is hard to understand why *T. forsythia* was not previously compared with *P. gingivalis* (Wegner et al., 2010) based on PAD type activity. All these results suggest that *T. forsythia* could express proteins with a PAD‐type capacity and therefore participate with *P. gingivalis* in establishing an oral microenvironment capable of citrullination. This could provide a logical explanation for studies where no association was observed between *P. gingivalis* and RA. In addition, and perhaps most importantly, these results may explain the differences between PAD activity and that dependent on the enzyme expressed by *P. gingivalis* (PPAD; Laugisch et al., 2016). In agreement with this, a recent study found that PAD activity in RA patients was increased in comparison to PPAD activity (Laugisch et al., 2016), suggesting other periodontal bacteria could participate in host protein citrullination.

Of note, this study has also shown a strong correlation between total ammonium levels in saliva and clinical RA activity. Although initially it was proposed that the enzymatic assays allowed for the evaluation of bacterial PAD activity, it is now evident that this technique does not distinguish if the ammonium is already present in the saliva sample, considering that the levels of ammonium in saliva depend, among other factors, on the blood ammonium content and the metabolism of urea and arginine by the oral microbiome (Adeva, Souto, Blanco, & Donapetry, 2012, Huizenga, Vissink, Kuipers, & Gips, 1999). In the present study, the patients were not selected but correspond to patients who attended the rheumatology clinic regularly and consecutively. Also, saliva samples were collected, stored, and analyzed under the same circumstances, so it could be suggested that the variations of total ammonium observed in the results depends fundamentally on the microbacterial component present in the patients. In that sense, the enriched presence of *T. forsythia* and *P. gingivalis* in patients with high RA activity seems to coincide with higher ammonium levels in the saliva samples. This same effect is observed when comparing the cultures the bacterial strains, where these two bacteria were the only ones that showed an increase in ammonium generation. We would primarily support the hypothesis that the increase in ammonium content in our enzymatic assay is a consequence of the action of PAD proteins, an event clearly demonstrated for *P. gingivalis* (Gabarrini et al., 2015, Montgomery et al., 2016); however, it remains to be evaluated in the case of *T. forsythia*. In any case, the results of this study suggest that the high prevalence of *T. forsythia* significantly alters the oral microenvironment and that this change seems to be related to the increased clinical activity in patients with RA. On the contrary, however, no association was found between total ammonium levels in saliva, bacteria prevalence and αCCP antibody titres, suggesting that the autoantibody repertoire induced by PD is more complex than expected or, as others have previously described, that the autoantibody repertoire is predominantly oriented to uncitrullinated peptides (De Pablo, P. et al. 2014).

Another important limitation of our study is the fact that the RA patients had different durations of disease‐modifying antirheumatic drug treatment, although no differences in treatment were evident between disease activity groups (Table 1). Another limiting parameter was the heterogeneous number of patients in the disease activity groups, where 23% of all patients showed low activity when compared to the high activity group (77%). With respect to PD, and per previous studies, a high percentage of Mexican RA patients fall in the group with severe PD (>80%). Finally, oral samples evaluated in this study were constituted by saliva, which could differ in protein and bacterial content from the gingival crevicular fluid.

In conclusion, our present results confirm that high RA activity is associated with severe PD, probing depth, high salivary total ammonium levels, and the presence of *T. forsythia* in the oral microenvironment, suggesting that other bacteria than *P. gingivalis* could be involved in the development of RA. The search for more bacteria and their association with RA are an important motive for improving care in oral hygiene and the quality of life of patients.

## CONFLICT OF INTEREST

None declared.

## SOURCE OF FUNDING

This work was supported by a grant from SEP/CONACYT CB‐2010 (#155392). J.I.M.R is a PhD student of the Graduate Sciences Program, Instituto Nacional de Salud Pública and was supported by a CONACYT grant (355015).

## CLINICAL RELEVANCE

### Scientific rationale for study

The role of periodontal bacterial citrullination (mediated by *Porphyromonas gingivalis*) is a risk factor for the development of autoantibodies in RA; however, this might not be an isolated event but part of a larger interaction between bacterium and other risk factors leading to autoimmunity.

### Principal findings

Disease activity in RA patients is associated with the presence of *Tannerella forsythia* and a higher ammonium levels in saliva, suggesting that this microorganism acts synergically with other risk factors in RA inflammation, including *P. gingivalis*.

### Practical implications

*P. gingivalis* and *T. forsythia* act synergically in RA patients leading to greater RA disease activity.

## Supporting information

Figure S1. Comparison of total ammonium levels between technique control and salivary samples of healthy donors or RA patients. Enzymatic assay for total ammonium levels determination is described in Methods. Graphical analysis was done without normalizing for experimental control (without oral samples). Statistical comparison was done employing Mann‐Whitney test, considering p<0.01 (**).

Figure S2. Salivary samples from patients with severe periodontitis showed higher total ammonium levels compared to those with mild periodontitis. Oral samples from RA patients were grouped per the degree of periodontitis into mild and severe. Comparing enzymatic total ammonium levels between these two groups, the severe periodontitis group showed higher total ammonium levels in comparison to the mild periodontitis group (p<0.01, **).
