# Supplementary figures and images for: Salivary ammonia levels and Tannerella forsythia are associated with rheumatoid arthritis: A cross sectional study
Source: Clin Exp Dent Res. 2017 Jun 7;3(3):107–14. doi: 10.1002/cre2.68 (PMC5719825; doi:10.1002/cre2.68)

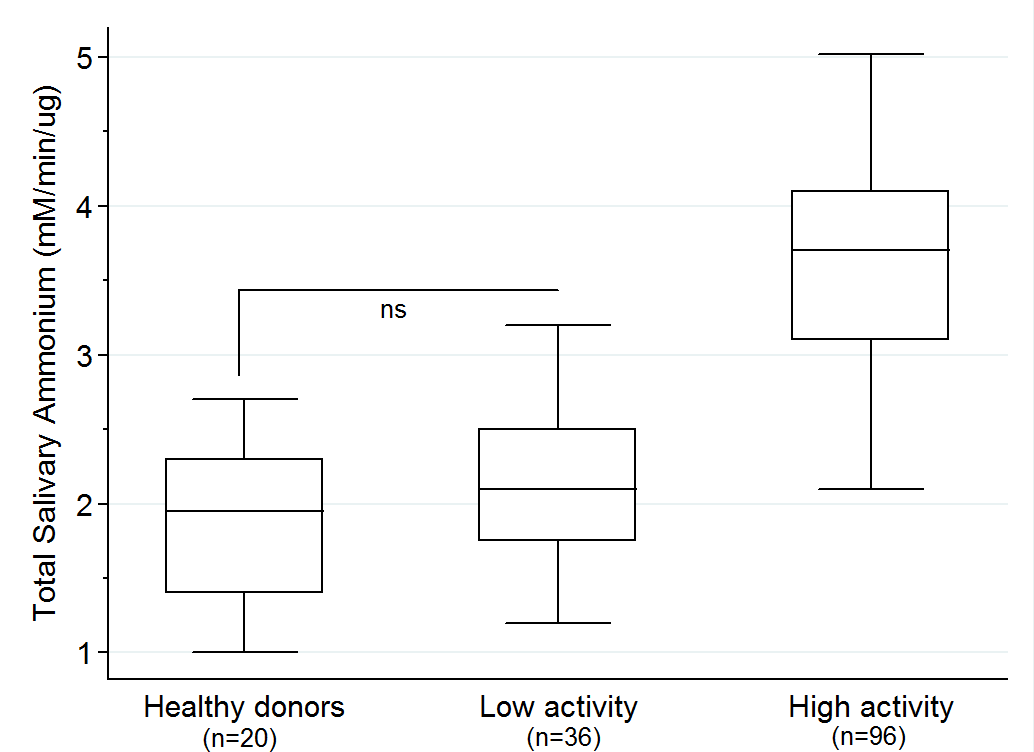

Supplement: Supplementary file 1 — Figure S1. Comparison of total ammonium levels between technique control and salivary samples of healthy donors or RA patients. Enzymatic assay for total ammonium levels determination is described in Methods. Graphical analysis was done without normalizing for experimental control (without oral samples). Statistical comparison was done employing Mann‐Whitney test, considering p<0.01 (**). [file CRE2-3-107-s001.tif]

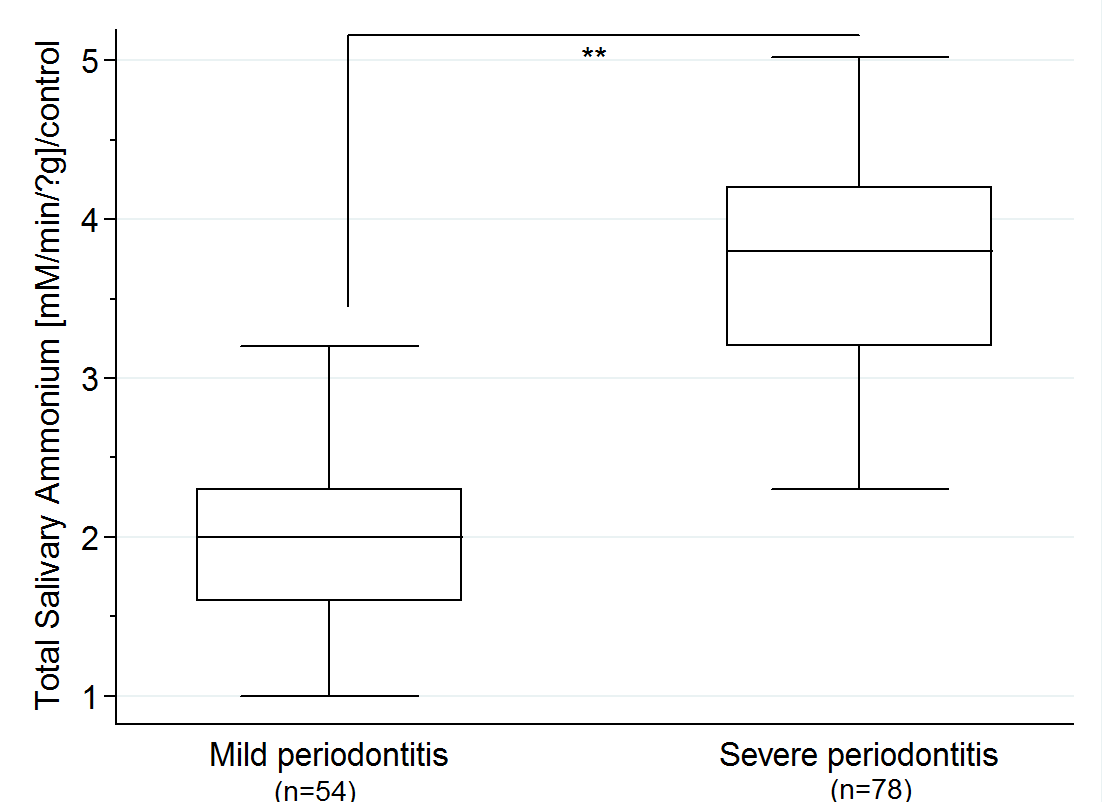

Supplement: Supplementary file 2 — Figure S2. Salivary samples from patients with severe periodontitis showed higher total ammonium levels compared to those with mild periodontitis. Oral samples from RA patients were grouped per the degree of periodontitis into mild and severe. Comparing enzymatic total ammonium levels between these two groups, the severe periodontitis group showed higher total ammonium levels in comparison to the mild periodontitis group (p<0.01, **). [file CRE2-3-107-s002.tif]
